# Development of a holistic communication score (HoCoS) in patients treated for oral or oropharyngeal cancer: Preliminary validation

**DOI:** 10.1111/1460-6984.12766

**Published:** 2022-08-31

**Authors:** Mathieu Balaguer, Julien Pinquier, Jérôme Farinas, Virginie Woisard

**Affiliations:** ^1^ IRIT Université de Toulouse, CNRS, Toulouse INP, UT3 Toulouse France; ^2^ Hôpital Larrey Hôpitaux de Toulouse Toulouse France; ^3^ Laboratoire de Neuro‐Psycho‐Linguistique LNPL Université Toulouse II Toulouse France

**Keywords:** assessment, communication, oncology, speech

## Abstract

**Background:**

In head and neck cancer, many tools exist to measure speech impairment, but few evaluate the impact on communication abilities. Some self‐administered questionnaires are available to assess general activity limitations including communication. Others are not validated in oncology. These different tools result in scores that does not provide an accurate measure of the communication limitations perceived by the patients.

**Aim:**

To develop a holistic score measuring the functional impact of speech disorders on communication in patients treated for oral or oropharyngeal cancer, in two steps: its construction and its validation.

**Methods & Procedures:**

Patients treated for oral/oropharyngeal cancer filled six self‐questionnaires: two about communicative dynamics (ECVB and DIP), two assessing speech function (PHI and CHI) and two relating to quality of life (EORTC QLQ‐C30 and EORTC QLQ‐H&N35). A total of 174 items were initially collected. A dimensionality reduction methodology was then applied. Face validity analysis led to eliminate non‐relevant items by surveying a panel of nine experts from communication‐related disciplines (linguistics, medicine, speech pathology, computer science). Construct validity analysis led to eliminate redundant and insufficiently variable items. Finally, the holistic communication score was elaborated by principal component factor and validated using cross‐validation and latent profile analysis.

**Outcomes & Results:**

A total of 25 patients filled the questionnaires (median age = 67 years, EIQ = 12; 15 men, 10 women; oral cavity = 14, oropharynx = 10, two locations = 1). After face validity analysis, 44 items were retained (*κ* > 0.80). Four additional items were excluded because of a very high correlation (*r* > 0.90) with other items presenting a better dispersion. A total of 40 items were finally included in the factor analysis. A post‐analysis score prediction was performed (mean = 100; SD = 10). A total of 24 items are finally retained for the construction of the holistic communication score (HoCoS): 19 items from questionnaires assessing communicative dynamics (13 from the ECVB and six from the DIP), four items from a perceived speech impairment questionnaire (PHI) and one from a quality‐of‐life questionnaire (EORTC QLQ‐H&N35). The reliability is good (five‐fold cross‐validation: *r*_s_ = 0.91) and the complementary latent profile analysis shows a good validity of the HoCoS, clustering subjects by level of communication performance.

**Conclusions & Implications:**

A global score allowing a measure of the impact of the speech disorder on communication was developed. It fills the lack of this type of score in head and neck oncology and allows the better understanding of the functional and psychosocial consequences of the pathology in the patients’ follow‐up.

**What this paper adds:**

**What is already known on the subject**

- Because of their anatomical location, head and neck cancer degrades the speech abilities. Few tools currently allow the assessment of the impact of the speech disorder on communication abilities. In ENT oncology, self‐administered questionnaires are available to assess activity limitations and participation restrictions (International Classification of Functioning (ICF)—WHO). Other tools from the field of neurology allow an evaluation of communication dynamics. But these different tools, constructed by items, give global additive or averaged scores. This implies an identical weighting of each item, resulting in global scores that are not very representative of the communication limitations really perceived by the patients.

**What this paper adds to existing knowledge**

- A new global holistic score allowing a measurement of the impact of speech impairment on communication after treatment of oral or oropharyngeal cancer has been developed. The methodology of its construction allows a better reflection of the symptomatological, pragmatic and psychosocial elements leading to a degradation of communication abilities.

**What are the potential or actual clinical implications of this work?**

- The developed HoCoS score fills the gap in the absence of this type of tool in head and neck oncology. It may allow a better understanding of the factors involved in the functional and psychosocial limitations of these patients, and better customize their follow‐up.

## INTRODUCTION

Cancer of the oral cavity and oropharynx is very common, with a high incidence: they represent more than 475,000 new cases worldwide in 2020.^1^ At the same time, mortality from lip–mouth–pharynx cancer is decreasing. Thus, the increase in life expectancy following cancer means that patients are now living longer with the sequelae of cancer and treatment (Borggreven et al., 2007).

In this context, the functional and psychosocial repercussions after oncological treatment must be considered, alongside the analytical and dynamic deficits.

For this purpose, various conceptual frameworks, based on bio‐psychosocial models, have been developed. The International Classification of Functioning, Disability and Health (ICF) developed by the World Health Organization (WHO) (2001) suggests looking beyond the impairment to the functional (activity limitations) and psychosocial consequences (participation restrictions) of pathologies. In this classification, personal and environmental factors can impact functional and psychosocial levels. These models provide a better understanding of the impact of therapeutic procedures in head and neck cancer on patients’ quality of life (Borggreven et al., 2007).

Complementary conceptual models have been described to specify the different levels involved in the functional and psychosocial dynamics, and to establish the causal relationships that may exist between them (Wilson & Cleary, 1995): biological and physiological factors, symptomatic status, functional status, general health perceptions and overall quality of life (Murphy et al., 2007).

Due to their location, oral and oropharyngeal cancer impact the speech abilities (Balaguer et al., 2019, 2020; Mlynarek et al., 2008), and are a common complaint of these patients. This symptomatology influences functional status, altering the patients’ communication abilities (Eadie et al., 2018). Yet, while many perceptual and automatic tools currently exist in head and neck oncology to measure speech impairment (Middag et al., 2009; Woisard et al., 2021) few assess the functional impact on communication abilities (Bolt et al., 2016; Meyer et al., 2004).

Some questionnaires assess activity limitations and participation restrictions, such as the phonation handicap index (PHI) (Balaguer et al., 2020) or the ‘phonation’ or ‘psychosocial’ domains of the carcinologic handicap index (CHI) (Balaguer et al., 2021). Other items related to communication, social contact or speech are present in both modules of the European Organization for Research and Treatment of Cancer (EORTC) quality of life (QoL) questionnaires (Aaronson et al., 1993; Bjordal et al., 1999).

Other questionnaires assess communication function but are not validated in head and neck oncology, such as the ECVB (Échelle de Communication Verbale de Bordeaux) (Mazaux et al., 2006) and the dysarthria impact profile (DIP) (Letanneux et al., 2013; Walshe et al., 2009). Some others target the assessment of communication abilities but present a very short format limiting the comprehensiveness of the assessment of the communication situation, for example, the Communicative Participation Item Bank (CPIB) (Baylor et al., 2009).

Moreover, the scoring of communication impairment is a crucial matter in the development of these tools. Indeed, these questionnaires result in scores per item, grouped in global scores. Because of their construction, by addition or average, these scores and subscores relating to a functional dimension make the hypothesis that each item carries the same weight in the construction of the final communication score. However, this strong assumption is difficult to support in a clinical setting because of the specific perceptions of each patient of their communication capacity. Moreover, all these questionnaires target different aspects of communication, but none of the tools allows to obtain a global, holistic score, representative of the impact on communication of speech disorders in head and neck oncology.

The objective of the present study is to develop a holistic communication score measuring the functional impact of speech disorders on communication in patients treated for oral or oropharyngeal cancer. The development of this score includes two sub‐objectives: its construction and its validation.

## METHODS

### Design

This is a cross‐sectional observational study.

The study protocol was approved by the Committee for the Protection of Persons (CPP: Ouest IV, 19 February 2020, reference 11/20_3) within the framework of the ANR RUGBI project.^2^

### Participants

Patients coming for consultation or hospitalization in an ear, nose and throat (ENT) service were recruited by the medical staff.

Inclusion criteria were: being of legal age (at least 18 years old) and having been treated for cancer of the oral cavity or oropharynx (surgical treatment and/or radiotherapy and/or chemotherapy) for at least 6 months (stable disorders). Patients with any other associated chronic disease were excluded.

All subjects who could be included during the inclusion period (October 2019–December 2020) were asked to participate in this study. The inclusion period corresponds to the inclusion period of the quality‐of‐life work package of the main study (RUGBI project).

### Selection process of questionnaires

#### Communication‐related questionnaires

Two questionnaires relating to communicative dynamics were retained in their entirety because of their comprehensiveness construction and their conceptual proximity to our objective of measuring the alteration of communicative abilities: the ECVB (Mazaux et al., 2006) and the DIP (Letanneux et al., 2013; Walshe et al., 2009). These questionnaires are only validated in an adult population with neurological pathologies. Nevertheless, they were retained in the constitution of the corpus of this study because they allow an ecological measurement (i.e., close to the real situations of real daily life) of communication, while taking into account the psycho‐affective dimension.

The ECVB (Mazaux et al., 2006) includes 34 items divided into seven dimensions corresponding to daily communication situations: expression of intentions (three items), conversation (seven items), telephone (seven items), shopping (four items), social relations (five items), reading (four items) and writing (four items). Initially validated with stroke patients, the dimensions assessed concern all aspects of communication that may be impaired, whether oral or written. Each item is rated on the principle of a Likert scale with four levels (‘never’, ‘rarely’, ‘often’ and ‘very often’, from 0 to 3 for questions with a positive polarity, and from 3 to 0 for negative ones). The lower the scores, the greater is the discomfort. In this study, this questionnaire was filled by the patient himself, as it is usually done in the patients reported outcomes (PRO) questionnaires (Doward & McKenna, 2004).

The DIP, initially validated in English (Walshe et al., 2009), has been translated and validated in French (Letanneux et al., 2013). Intended for subjects with Parkinson's disease, it includes 48 items in four dimensions: ‘the effect of dysarthria on me as a person’ (12 items), ‘accepting my dysarthria’ (10 items), ‘how I feel others react to my speech’ (14 items), and ‘how dysarthria affects my communication with others’ (12 items). These 48 items are also presented in the form of a five‐level Likert scale (strongly disagree, agree, not sure, disagree, strongly disagree: the direction of the scoring depending on the polarity of the questions). For this study, we added an item to section ‘how I feel others react to my speech’ to clarify an abstruse French formulation. The DIP therefore includes here 49 items.

#### Questionnaires assessing speech function

Some questionnaires used in the routine care include items relating to the functional or psychosocial consequences of the speech disorder in head and neck cancer: the phonation handicap index (PHI) (Balaguer et al., 2020) and the Carcinologic handicap index (CHI) (Balaguer et al., 2021).

Focusing on speech self‐assessment, the PHI was retained in its entirety: five items in the physical signs (PHI‐F) domain, five items in the functional impact (PHI‐C) domain, five items of the psychosocial repercussions domain (PHI‐E), and three complementary questions (What degree of severity do you give to your speech difficulties?; How difficult is it for you to produce understandable speech?; and How much does your speech impairment affect your daily life?’).

Allowing a measure of patients’ needs after cancer treatment, the CHI also includes dimensions and items related to speech: four items in the dedicated phonation domain, and four items of the psychosocial domain.

#### Quality of life (QoL) questionnaires

Finally, other questionnaires are designed to measure psychosocial consequences in terms of impact on QoL. The EORTC reference questionnaires were retained for further analyses: 30 items of the EORTC QLQ‐C30 (Aaronson et al., 1993) and 35 of the EORTC QLQ‐H&N35 (Bjordal et al., 1999) measuring global and speech‐related quality of life.

### Construction of the score

Characterization of the variable type for the holistic communication score (HoCoS)

The determination of the type of the targeted variable is essential because it will condition the statistical methodology used in dimensionality reduction (Carreira‐Perpinán, 1997; Chadeau‐Hyam et al., 2013; Cunningham, 2014). This methodology is required due to the format of the data, where a large number of items (*n* = 174) was retained.

The holistic communication score (HoCoS) was considered as a latent variable (Borsboom, 2008): it cannot be directly observed or measured, and it requires several manifest variables as indicators that are observable and measurable. In our study, the latent HoCoS influences the values of the measured manifest variables, that is, the items from the different questionnaires. Although the responses to these variables are constructed in the form of Likert scales (i.e., ordinal categorical), they were treated in this study according to a naive approach, that is, as quantitative variables. A Shapiro–Wilk test shows that less than a quarter of these manifest variables do not have a normal distribution, which supports the choice of this approach. The HoCoS was thus elaborated as a quantitative latent variable.

#### Face validity

A panel of nine experts from different communication‐related disciplines was surveyed to define the criteria for inclusion of items in the HoCoS. All the experts (two computer scientists, two speech and language pathologists and PhD students, two speech therapists, one phoniatrician, two researchers in linguistics) were involved for at least 5 years in several research projects related to pathological speech analysis. Moreover, all the speech therapists currently worked in ENT departments.

This selection of relevant items was performed in two steps. First, the experts were surveyed to get a consensus definition of the communication abilities in the context of oral or oropharyngeal cancer. According to the consensual definition, the items from the questionnaires that did not comply with this consensus definition were removed. The experts then participated to an individual selection of the items.

Items were finally retained if they met one of the following two criteria:

- The I‐CVI (item‐level content validity index) (Lynn, 1986; Polit et al., 2007) was >0.777, which corresponds to an agreement of seven out of nine experts to keep the item.
- The Kappa of agreement was ≥0.81 (Landis & Koch, 1977): ‘almost perfect’ agreement.

#### Construct validity

A statistical selection of items respecting face validity was carried out on the criteria of non‐redundancy and sufficient variability.

The criterion of non‐redundancy allows an analysis if the scores of the items are statistically not associated with each other. An analysis of the inter‐item correlation matrix using Spearman coefficients (non‐parametric) was used because of the small sample size (*n* < 30). A threshold of 0.90 was chosen: only one of the items correlated with each other at ≥0.90 could be retained for further analysis.

In this case, to obtain sufficiently variable items, and thus allowing a more specific measure of inter‐individual variability, only the item with the highest coefficient of variation (if the distribution of this item is gaussian, tested by the Shapiro–Wilk test) or the highest dispersion index (if the distribution is not gaussian) was retained.

#### Elaboration of the holistic communication score (HoCoS)

Once relevant, non‐redundant and sufficiently variable items were selected, the HoCoS was elaborated, using principal component factor (PCF) analysis (Roscoe et al., 1982). PCF analysis is commonly used in data reduction (Acock, 2018) because it attempts to explain as much as possible the variance of a set of items by a single dimension, in other words when a set of items all measure the same concept (communalities set to one, no uniqueness).

This statistical technique therefore suits the objective of this study, where a single quantitative latent holistic score (the HoCoS) is sought among the set of manifest variables (corresponding to the selected items).

Thus, a prediction of the values in PCF analysis for factor 1, corresponding to the latent variable HoCoS, was made. This prediction is derived from a regression analysis on the set of new variables created by estimation of the first factor.

Thus, per subject, the score is predicted by the sum of the standardized values of each item weighted by the regression coefficient corresponding to factor 1.

### Validation of the HoCoS

The validation of the score was done in two steps. First, a five‐fold cross‐validation of the predicted score was led to verify the reliability of the score. A latent profile analysis (Cai, 2012) leading to the construction of a qualitative HoCoS (‘HoCoS‐Qual’) was then compared with the (quantitative) HoCoS.

This type of analysis, based on generalized structural equation modelling (GSEM) models (Coma et al., 2013), allows one to determine which individuals are most likely to belong to a group (corresponding to a category of the latent variable) according to information carried by other variables. Two‐, three‐ and four‐class models were computed, and the model with the best Akaike information criterion (AIC) and Bayesian information criterion (BIC) criteria—i.e., the lowest parameters—was selected (Cameron & Trivedi, 2005).

Finally, the class of each subject was predicted. For each subject, the categorical latent variable HoCoS‐Qual thus takes one of the values corresponding to one of the classes.

### Statistical analyses

The statistical analyses were carried out using Stata 16.1 software (StataCorp. 2019. Stata Statistical Software: Release 16. College Station, TX, USA: StataCorp LLC).

In all analyses, a level of significance at 5% was chosen.

The normality of distribution of quantitative variables was tested using a Shapiro–Wilk test.

Correlation analyses were performed using the following thresholds (Mukaka, 2012): >0.9 (very high correlation), 0.7–0.9 (high correlation), 0.5–0.7 (moderate correlation), 0.3–0.5 (low correlation), and <0.3 (negligible correlation).

## RESULTS

### Participants

A total of 25 patients filled the questionnaires (median age = 67 years, IQR = 12; 15 males and 10 females; oral cavity = 14, oropharynx = 10, two locations = 1).

A total of 88% of the subjects were treated surgically, 96% by radiotherapy.

The mean time after treatment was 87.2 months (SD = 121.8; median = 40; interquartile range = 123).

Details of TNM classification and treatment strategies are given in Table 1.

**TABLE 1:** Characteristics of the subjects

	**Total (%)**	
*TNM Classification: T (tumour size)*	25	
0	0	0.0%
1	2	10.5%
2	6	31.6%
3	3	15.8%
4	8	42.1%
Missing data	6	
*Treatments*	25	
Surgery alone	1	4%
Radiotherapy alone	1	4%
Surgery + radiotherapy	8	32%
Surgery + radiotherapy + chemotherapy	13	52%
Radiotherapy + chemotherapy	2	8%
*Speech and language therapy*	**25**	
Ongoing	12	55%
Once a week	*3*	
At least twice a week	*3*	
No information	*6*	
None	10	45%
Missing data	3	

### Construction of the HoCoS

A set of 174 items were initially collected. The list of the 174 items and reasons for exclusion (if applicable) are given in in the additional supporting information.

**FIGURE 1:**
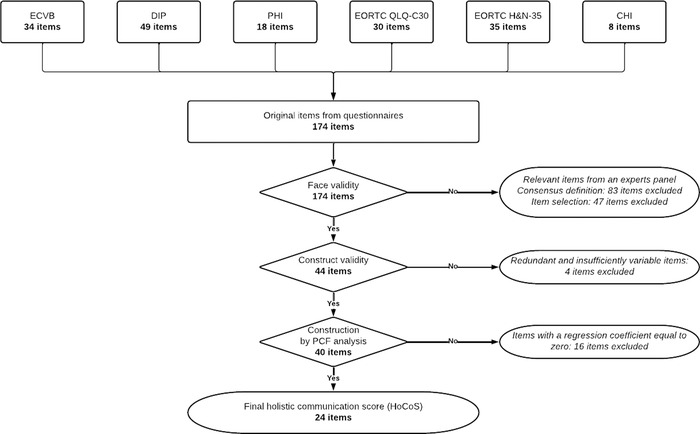
Construction process of the holistic communication score

The construction process is represented in Figure 1 and will be developed below.

#### Face validity

As a first step, the panel identified the following consensus criteria for retaining items:

- Items related to oral communication: exclusion of items related to written communication unless they allow for compensation or an increase in oral communication.
- Items relating to expression: exclusion of only comprehension‐related items.
- Items relating to interaction between speaker and interlocutor, even if implicit.

This first step led to exclude 83 items: those relating to reading, writing, understanding conversation, items relating to fatigue, pain, specific speech symptoms such as speed of speech, etc.

A total of 91 items (52.3%) were thus retained for further analysis.

In a second step, an online questionnaire using the LimeSurvey tool was submitted to the nine experts. They were asked to indicate which of the remaining 91 items should be retained in the elaboration of the holistic communication score. The experts had to tick the boxes corresponding to all the items to be kept.

**FIGURE 2:**
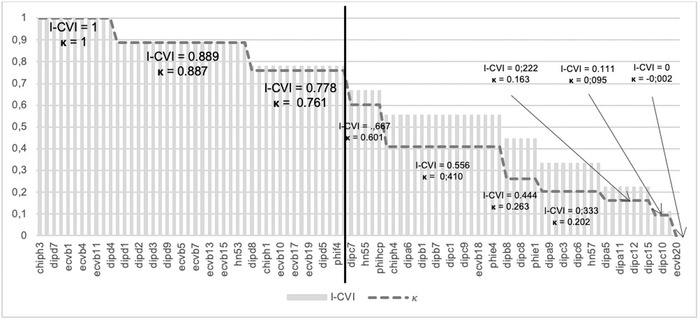
I‐CVI scores and Kappa of agreement, with cut‐off (black line)

A total of 44 items (48.4%) met one of the two previously defined conditions (I‐CVI > 0.777 or *κ* ≥ 0.81) and were retained for further analysis (Figure 2).

#### Construct validity

Four items were excluded because of a too high correlation with other items (considered as redundant items). In that case, only the item with the bigger variation coefficient or dispersion index was retained (Table 2).

**TABLE 2:** Non‐redundancy analysis

**Correlation between the two items of the pair**	**Item code**	**Item**	**Variation coefficient^a^ or dispersion index^b^**
0.95	ecvb11	Do you have difficulty calling your family?	0.67^a^
	**ecvb12**	**Do you have difficulty phoning your friends?**	**0.71^a^**
0.92	chiph3	Do you speak less with your family, friends, neighbours?	0.77^a^
	**phie2**	**My speech difficulties limit my personal and social life**	**0.84^a^**
0.91	**ecvb8**	**And with someone you don't know very well (the letter carrier or a cab driver for example), are you embarrassed to have a conversation on simple subjects? (The weather; what you did the day before; the flowers in your garden …)?**	**0.87^b^**
	ecvb10	Do you find it difficult to speak when you are with people you don't know well (at a dinner party, an outing, an evening out …)?	0.53^a^
0.90	chiph2	Do people have difficulty understanding you?	0.61^a^
	**phic4**	**I am asked to repeat myself because of my difficulty to speak**	**0.69^a^**

*Note*: Retained items are shown in bold.

A total of 40 items were finally retained for the construction of the holistic communication score.

#### Elaboration of the holistic communication score (HoCoS)

The PCF analysis has two conditions of application: first, the first main factor must explain a ‘substantial’ part of the total variance for all the items; and second, most of the items must have a load of ≥0.4 on this factor.

**FIGURE 3:**
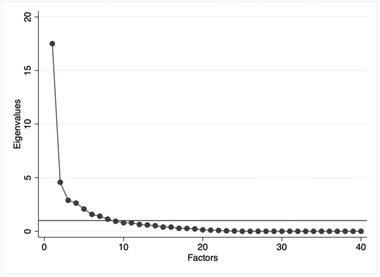
Scree plot of the eigenvalues of the factors following the PCF analysis

Both conditions are met. The eigenvalue of factor 1 (i.e., the proportion of total variance attributable to factor 1) is 17.51 compared with 4.57 for factor 2 (Figure 3). Moreover, the proportion of variance explained solely by factor 1 is 0.44 (0.11 for factor 2). Finally, 34 out of 40 items (85%) have a loading of ≥0.4 on this factor.

Since PCF analysis is applicable, a prediction of values for factor 1, corresponding to the latent variable HoCoS, was performed.

A total of 16 items have a coefficient equal to zero, neutralizing the value taken by the item, and thus indicating that they will not be considered in the overall calculation of the HoCoS. Therefore, 24 items out of 40 (60%) have non‐zero coefficients and will be retained for the HoCoS calculation.

To facilitate readability and interpretation, the predicted score, initially centred on zero and having a standard deviation (SD) of 1, was centred on 100 with a SD of 10. This transformed score constitutes the HoCoS.

The HoCoS is therefore calculated as follows:

$$\begin{array}{ccc} HoCoS & & =\left(\frac{\left[\sum \left(\frac{X_{item}-m_{item}}{s_{item}}\times \beta_{item}\right)\right]-m_{initial\_score}}{s_{initial\_score}}\times 10\right)\\ & & +100\\ & & =\left(\frac{\left[\sum \left(\frac{X_{item}-m_{item}}{s_{item}}\times \beta_{item}\right)\right]-\;0.0353408}{0.999787}\times 10\right)\\ & & +100 \end{array}$$

where *X*_item_ represents the raw item value (score obtained on the item by the subject); *m*_item_ is the mean of the item obtained in the study sample, *s*_item_ is the SD of the item obtained in the study sample, *β*_item_ represents the regression coefficient of the item; *m*_initial_score_ is the mean of the initially predicted score; and *s*_initial_score_ is the SD of the initially predicted score.

**FIGURE 4:**
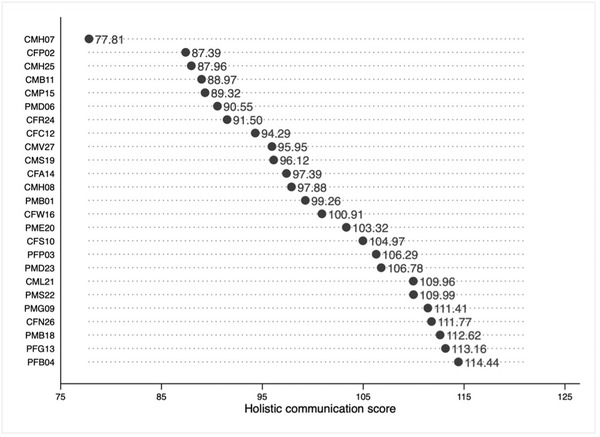
Holistic communication scores (HoCoS) by subject

The HoCoS are shown in Figure 4.

The complete formula is as follows:

$$\begin{array}{ccc} HoCoS & = & (((\left(\left(ecvb1-2.36\right)\;/0.8103497^{*}0.03264\right)\\ & & +\left(\left(ecvb4-2.08\right)\;/0.9539392^{*}0.01566\right)\\ & & +\;\left(\left(ecvb5-1.8\right)\;/0.9128709^{*}0.18626\right)\\ & & +\left(\left(ecvb6-1.4932\right)\;/0.8338601^{*}0.10411\right)\\ & & +\left(\left(ecvb7-.34\right)\;/0.8524999^{*}0.01695\right)\\ & & +\left(\left(ecvb9-1.8\right)\;/0.9574271^{*}-0.00532\right)\\ & & +\;\left(\left(ecvb12-1.68\right)\;/1.215182^{*}-0.01413\right)\\ & & +\left(\left(ecvb13-1.56\right)\;/1.356466^{*}0.10219\right)\\ & & +\left(\left(ecvb16-1.72\right)\;/1.137248^{*}0.12197\right)\\ & & +\;\left(\left(ecvb17-2.3268\right)\;/0.8902^{*}0.06856\right)\\ & & +\left(\left(ecvb19-1.4932\right)\;/1.054509^{*}-0.01007\right)\\ & & +\left(\left(ecvb25-1.82\right)\;/1.081376^{*}0.15096\right)\\ & & +\left(\left(ecvb26-2.28\right)\;/0.9363048^{*}-0.08137\right)\\ & & +\left(\left(dipd2-2.88\right)\;/1.563117^{*}-0.04681\right)\\ & & +\left(\left(dipd3-2.2\right)\;/0.8660254^{*}0.1402\right)\\ & & +\left(\left(dipd5-3.36\right)\;/1.113553^{*}-0.03138\right)\\ & & +\left(\left(dipd6-3.48\right)\;/1.262273^{*}0.14321\right)\\ & & +\;\left(\left(dipd7-2.64\right)\;/1.254326^{*}0.05331\right)\\ & & +\left(\left(dipd9-2.84\right)\;/1.344123^{*}-0.12441\right)\\ & & +\;\left(\left(hn53-2.2\right)\;/1^{*}-0.071\right)\\ & & +\left(\left(phif4-2.32\right)\;/1.519868^{*}-0.00858\right)\\ & & +\left(\left(phic1-0.99\right)\;/1.251^{*}-0.24677\right)\\ & & +\;\left(\left(phic3-1.76\right)\;/1.422439^{*}-0.08623\right)\\ & & +\left(\left(phic4-1.68\right)\;/1.180395^{*}-0.07575\right))\\ & & -\;0.0353408)\;/0.999787{}^{*}10)+100 \end{array}$$

### Validation of the HoCoS

#### Five‐fold cross‐validation

**FIGURE 5:**
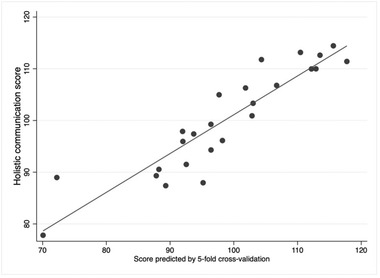
Scatterplot representing HoCoS and score predicted by cross‐validation, with regression line

A strong correlation of 0.91 was found between the HoCoS and the values predicted by the five‐fold cross‐validation (i.e., training on 20 observations and prediction on the other five observations, repeated five times in this case) (Figure 5).

#### Complementary validation by latent profile analysis

The same 40 items meeting the face and construct validities were retained as manifest variables of the qualitative latent score (HoCoS‐Qual).

The model selection parameters AIC and BIC were calculated for models with two, three and four classes (Table 3). The model resulting in a three‐class latent variable was thus retained because it has the lowest AIC and BIC criteria.

**TABLE 3:** Akaike information criterion (AIC) and Bayesian information criterion (BIC) values of GSEM models (latent profile analysis—LPA)

**Model**	**AIC**	**BIC**
Latent variable with two classes	2811.97	2959.45
**Latent variable with three classes**	**2729.59**	**2927.04**
Latent variable with four classes	2747.96	2995.39

*Note*: The retained model is shown in bold.

The class to which each subject belongs was then predicted. For each subject, the categorical latent variable HoCoS‐Qual thus takes one of the three values corresponding to one of the three classes (1, 2 or 3).

A comparison of the values of the two latent variables, quantitative (reference HoCoS score) and categorical (HoCoS‐Qual), shows that class 1 of the HoCoS‐Qual score corresponds to the subjects with the lowest HoCoS scores, class 2 corresponds to the subjects with the highest HoCoS scores and finally class 3 to the subjects with intermediate HoCoS scores (Table 4).

**TABLE 4:** Comparison of latent classes predicted by latent profile analysis (LPA) analysis and the HoCoS score (subjects are ranked by increasing order of the HoCoS)

**Subject**	**Predicted class**	**HoCoS**	**Subject**	**Predicted class**	**HoCoS**	**Subject**	**Predicted class**	**HoCoS**
CMH07	1	77.81	CMV27	3	95.95	CFS10	2	104.97
CFP02		87.39	CMS19		96.12	PFP03		106.29
CMH25		87.96	CFA14		97.39	PMD23		106.78
CMB11		88.97	CMH08		97.88	CML21		109.96
CMP15		89.32	PMB01		99.26	PMS22		109.99
PMD06		90.55	CFW16		100.91	PMG09		111.41
CFR24		91.50	PME20		103.32	CFN26		111.77
CFC12		94.29				PMB18		112.62
						PFG13		113.16
						PFB04		114.44

The latent profile analysis leads to a qualitative variable corresponding to a level of impact on communication closely related to the HoCoS score. This analysis also confirms that the HoCoS does correspond to a level of impact on communication, thus validating the construction of this holistic indicator.

## DISCUSSION

### Psychometrics

A new index measuring communication impairment in patients treated for oral or oropharyngeal cancer was developed in this study.

Despite its construction from limited sample (*n* = 25), the HoCoS shows good performances in validity and reliability. On the one hand, the reliability in cross‐validation is high (*r*_S_ = 0.91). On the other hand, it is also a valid score, which measures the level of impact on communication, which was confirmed by the construction of a qualitative index by a latent profile analysis.

### Limitations

However, to better ensure generalizability of the results, this study needs to be completed.

First, increasing the sample size would allow a better statistical power and thus a better generalization of the results. Thus, this study concerns the first validation step of this innovative tool. This preliminary study will have to be followed by a validation of the HoCoS on a larger sample size. To date, there is no consensus on the number of subjects required for score validation. The inclusion of a new sample of about 100 subjects could thus be considered to ensure greater statistical robustness.

Then this score should be evaluated on a new sample of patients for external validation. The analysis of the performance of the HoCoS on a new sample of patients would allow to ensure its reliability, and thus again its generalizability.

Finally, the temporal reliability of this score remains to be analysed. This point is closely related to the temporal reliability performance of the questionnaires from which the items used to calculate the HoCoS are taken. However, the non‐preservation of all the items of the initial questionnaires modifies the global structure of these questionnaires, and the temporal reliability remains to be verified on specific items presented in a different order. The 24 items retained for the construction of the HoCoS will thus have to be presented in two stages (D0 and D7) to a new sample of patients.

### Perspectives

#### Speaker and interlocutor in communication situation

The holistic communication score is elaborated solely from items from self‐reported questionnaires. The measure of communication impairment is therefore only self‐reported.

However, when measuring communication abilities, the speaker is a communication partner in the same way as the interlocutor. The latter is particularly important because communication is only effective if the message is not only correctly transmitted but also correctly received.

The inclusion in the HoCoS of an external measure of communication abilities by a listener would allow to consider other dimensions linked more globally to the impact of the disorder on the comprehensibility of speech (by a listener) (Pommée et al., 2021) and by ripple effect on communication and quality of life. Different tools could be used for this purpose, such as an evaluation by the listener using a visual analogue scale, or communicative dynamics questionnaires for example. The analysis of the results of a score combining internal and external measures according to the tools used could provide new insights into the dynamics of communication in a social context.

#### Communication environment according to the bio‐psychosocial models

Bio‐psychosocial models such as Wilson's (Wilson & Cleary, 1995) represent the links between symptomatic (speech disorders) and functional (communication) status. According to these models, factors related to the characteristics of the individual or the environment can influence both the speech disorder and the communication abilities.

Taking into account these factors, such as the cognitive and anxiety–depressive state (Böhm et al., 2016; Eadie et al., 2018) the constitution of social circles around the patient (Danker et al., 2010) or the patient's self‐perception of the speech impairment (Bolt et al., 2016) would allow a better understanding of the functional dynamics in patients treated for oral cavity or oropharyngeal cancer.

More globally, the association of the holistic communication score with indicators related to the individual and his environment and a measure of the speech disorder could also allow a more effective prediction of the psychosocial impact of speech disorders in these patients, and their quality of life.

#### Clinical implications

The HoCoS is an index that is voluntarily holistic in its construction, taking into account symptomatologic (e.g., item phif4 ‘I use a great deal of effort to speak’), interactional (e.g., item phic3 ‘I have trouble communicating with unfamiliar people’), pragmatic (e.g., ecvb25 ‘At the restaurant/coffee shop, do you find it difficult to place your order yourself?’), but also psycho‐affective (e.g., dipd9 ‘I feel comfortable speaking in most situations both at home and outside’) dimensions. Strategies for compensating for communication difficulties are also considered (e.g., dipd3 ‘I try other ways of getting my message across when people don't understand me’).

In our sample, 55% of the subjects included had speech therapy. However, speech therapy follow‐up can modify patients’ perception of their own communication abilities (Jacobi et al., 2010). The HoCoS would have a relevant clinical applicability in the rehabilitation process of head and neck cancer patients as one of the indicators to be considered by speech therapists, by providing a valid and reliable measure of progress in follow‐up.

This indicator thus fills a gap in tools for measuring the functional consequences of oral cavity and oropharyngeal cancer treatment on speech and communication in daily care. The use of the HoCoS in clinical care would allow to better target the daily problems met by the patients in their communication with peers, and thus to better adapt the therapeutic strategies to their reported needs.

It thus seems interesting to let the patient in head and neck oncology to fill the HoCoS, that is, the 24 items from the ECVB, DIP, PHI and EORTC QLQ‐H&N35 questionnaires. This score structured in few items allows to quickly target the impact of the speech disorder on communication. This measurement could be systematized in consultation or evaluation and would allow the therapeutic strategy to be adjusted as closely as possible to the patient's needs.

Finally, to consider the patients’ quality of life, beyond the functional dimension evaluated by our new score, the HoCoS could thus be associated with specific quality of life questionnaires (Raquel et al., 2020) in a bio‐psychosocial follow‐up approach.

## CONCLUSIONS

A global score allowing a measurement of the impact of speech impairment on communication after treatment of oral or oropharyngeal cancer has been developed. The methodology of its construction allows a better reflection of the symptomatological, pragmatic and psychosocial elements leading to a degradation of communication abilities. The temporal reliability of this score and its external validity remains to be explored. Nevertheless, it fills the gap in the absence of this type of tool in head and neck oncology, and may allow a better understanding of the factors involved in the functional and psychosocial limitations of these patients.

## CONFLICTS OF INTEREST

All authors declare that they have no conflict of interest.

## Supporting information



Supporting information

## Data Availability

Data and the database are available from the corresponding author upon request.

## NOTES

1 Source: Globocan—Global Cancer Observatory 2020 World Health Organization, https://gco.iarc.fr/today/data/factsheets/cancers/3‐Oropharynx‐fact‐sheet.pdf.

2 See https://www.irit.fr/rugbi/.
