# Supplementary material for: Development of a holistic communication score (HoCoS) in patients treated for oral or oropharyngeal cancer: Preliminary validation
Source: Int J Lang Commun Disord. 2022 Aug 31;58(1):39–51. doi: 10.1111/1460-6984.12766 (PMC10087239; doi:10.1111/1460-6984.12766)
Supplement: Supplementary file 1 — Supporting information [file JLCD-58-39-s001.docx]

**Supplementary material** Reasons for exclusion and retention of 174 items from the self-completed questionnaires

*The lines of the items selected for the HoCoS are in grey*

| **Code** | **Item** | **Not in accordance with the definition by the committee of experts** | **Not retained by the experts** | **Redundant / insufficiently variable** | **Regression coefficient equal to 0** |
| --- | --- | --- | --- | --- | --- |
| ecvb1 | Are you embarrassed to express very simple things, for example to say that you want to drink, to eat, to go to rest... ? (expression of a need) |  |  |  |  |
| ecvb2 | Are you embarrassed to express your desires, your intentions, for example to make it clear that you want to go for a walk (or) to see a particular person (or) to go to the hairdresser ...? (intention, project) |  |  |  | **1** |
| ecvb3 | When you go for a walk, when you go out alone, are you embarrassed to ask for directions? If the situation has not arisen, ask: are you embarrassed, for example, to explain your destination to a cab driver? |  |  |  | **1** |
| ecvb4 | With a family member or friend, do you have difficulty having a conversation about a common everyday topic (e.g., the weather, your favorite food, what you had for lunch...) |  |  |  |  |
| ecvb5 | What about a more abstract or complicated subject, for example: current events, education, unemployment, literature...? |  |  |  |  |
| ecvb6 | With family or friends, do you initiate the conversation? |  |  |  |  |
| ecvb7 | Do you have difficulty expressing feelings (e.g. joy, anger or fear) in words? |  |  |  |  |
| ecvb8 | And with someone you don't know very well (the letter carrier or a cab driver for example), are you embarrassed to have a conversation on simple subjects? (the weather; what you did the day before; the flowers in your garden...)? |  |  |  | **1** |
| ecvb9 | And more complicated topics, for example: do you have a problem explaining your situation or your illness to someone who doesn't know you? |  |  |  |  |
| ecvb10 | Do you find it difficult to speak when you are with people you don't know well (at a dinner party, an outing, an evening out...)? |  |  | **1** | **X** |
| ecvb11 | Do you have difficulty calling your family? |  |  | **1** | **X** |
| ecvb12 | Do you have difficulty phoning your friends? |  |  |  |  |
| ecvb13 | When you want to make an appointment with someone, for example your doctor or your speech therapist, is it you who calls? |  |  |  |  |
| ecvb14 | Are you embarrassed to call a stranger? For example calling a cab or calling the plumber in case of a water leak? |  |  |  | **1** |
| ecvb15 | When the phone rings and you are alone, do you answer it? |  |  |  | **1** |
| ecvb16 | And if there is someone else at home (spouse, child, friend...), do you answer the phone? |  |  |  |  |
| ecvb17 | Do you find it difficult to pass on a telephone message received in your absence to a loved one (spouse, child, neighbor, etc.)? |  |  |  |  |
| ecvb18 | Since your illness (or) our last interview (or) the beginning of rehabilitation (adapt as appropriate), have you gone shopping alone? (at least one: bread, lottery, bus ticket, newspaper, coffee or other consumption). |  | **1** | **X** | **X** |
| ecvb19 | In a store (outside of a department store) do you ask the salesperson what you want, if you can't find it? |  |  |  |  |
| ecvb20 | When you pay, do you have difficulty using cash, for example: giving the exact amount or counting your change? |  | **1** | **X** | **X** |
| ecvb21 | Do you use checks (or a credit card) to pay? | **1** | **X** | **X** | **X** |
| ecvb22 | At a family meal or with friends, do you find it difficult to follow a quick conversation between several people? | **1** | **X** | **X** | **X** |
| ecvb23 | When you need information, do you talk to strangers? You don't know his room number. Would you ask the receptionist?) |  |  |  | **1** |
| ecvb24 | Do you go out (to friends, to the movies, to restaurants, to the theater...)? |  | **1** | **X** | **X** |
| ecvb25 | At the restaurant/coffee shop, do you find it difficult to place your order yourself? |  |  |  |  |
| ecvb26 | When you go to a shop, for example to the grocer, the garage, the hairdresser, the bookstore... do you explain what you want? |  |  |  |  |
| ecvb27 | Do you read newspapers, magazines (possibly books)? | **1** | **X** | **X** | **X** |
| ecvb28 | Do you read the mail that your friends or family write to you? | **1** | **X** | **X** | **X** |
| ecvb29 | Do you have difficulty reading administrative papers or instructions? | **1** | **X** | **X** | **X** |
| ecvb30 | Do you have trouble telling time? | **1** | **X** | **X** | **X** |
| ecvb31 | Do you have difficulty writing single words (e.g., to-do list)? | **1** | **X** | **X** | **X** |
| ecvb32 | Do you have difficulty writing sentences (e.g., letter or card during vacation, you, birthday...)? | **1** | **X** | **X** | **X** |
| ecvb33 | Do you fill out social security forms or other administrative paperwork? | **1** | **X** | **X** | **X** |
| ecvb34 | Do you write checks? | **1** | **X** | **X** | **X** |
| dipa1 | My speech problem has had a negative effect on how I see myself | **1** | **X** | **X** | **X** |
| dipa2 | When I speak, I think I sound like somebody else, not me | **1** | **X** | **X** | **X** |
| dipa3 | Even when I am not speaking, I feel that I am a different person now | **1** | **X** | **X** | **X** |
| dipa4 | My speech does not make me feel inadequate |  | **1** | **X** | **X** |
| dipa5 | I am as confident now as I was before I had a speech problem |  | **1** | **X** | **X** |
| dipa6 | Because of my speech I am more dependent on people now than I was before |  | **1** | **X** | **X** |
| dipa7 | My speech does not make me feel self-conscious |  | **1** | **X** | **X** |
| dipa8 | My speech problem does not make me feel incompetent |  | **1** | **X** | **X** |
| dipa9 | I do not feel foolish when I am misunderstood |  | **1** | **X** | **X** |
| dipa10 | I feel stupid when someone asks me to repeat |  | **1** | **X** | **X** |
| dipa11 | I feel less in control of my life now because of my speech |  | **1** | **X** | **X** |
| dipa12 | My speech difficulty has not changed me fundamentally as a person | **1** | **X** | **X** | **X** |
| dipb1 | I do not try to hide my speech problem |  | **1** | **X** | **X** |
| dipb2 | I get mad when people do not understand me |  | **1** | **X** | **X** |
| dipb3 | I am not happy with my speech as it is now | **1** | **X** | **X** | **X** |
| dipb4 | I am sensitive about my speech |  | **1** | **X** | **X** |
| dipb5 | I do not worry about my speech |  | **1** | **X** | **X** |
| dipb6 | It does not bother me to admit that I have a speech problem |  | **1** | **X** | **X** |
| dipb7 | I would rather miss out on conversation than admit to having a speech problem |  | **1** | **X** | **X** |
| dipb8 | My speech has affected my life more than anything else has |  | **1** | **X** | **X** |
| dipb9 | I do not get angry when I cannot make myself understood |  | **1** | **X** | **X** |
| dipb10 | I have many other qualities besides speech that are important | **1** | **X** | **X** | **X** |
| dipc1 | I am conscious of other people’s reactions to my speech |  | **1** | **X** | **X** |
| dipc2 | My family never get annoyed when they do not understand what I am saying |  | **1** | **X** | **X** |
| dipc3 | I don’t care what people think of my speech |  | **1** | **X** | **X** |
| dipc4 | People treat me as if I am stupid because they can’t understand me |  | **1** | **X** | **X** |
| dipc5 | People don’t get embarrassed when I can’t make myself understood |  | **1** | **X** | **X** |
| dipc6 | Strangers do not treat me negatively because I have a speech problem |  | **1** | **X** | **X** |
| dipc7 | People are usually patient when I speak slowly |  | **1** | **X** | **X** |
| dipc8 | My friends make more of an effort to understand me than my family does |  | **1** | **X** | **X** |
| dipc9 | People pretend to understand what I have said when I know they haven’t |  | **1** | **X** | **X** |
| dipc10 | People sometimes think I have been drinking when I haven’t |  | **1** | **X** | **X** |
| dipc11 | Most people make an effort to understand what I am saying |  | **1** | **X** | **X** |
| dipc12 | Other peoples’ opinions of my speech matter to me |  | **1** | **X** | **X** |
| dipc13 | I never feel that others laugh or make fun of my speech |  | **1** | **X** | **X** |
| dipc14 | People are more condescending to me now because of my speech |  | **1** | **X** | **X** |
| dipc15 | Strangers don't have negative preconceived ideas about me despite my speech problem |  | **1** | **X** | **X** |
| dipd1 | Although I have difficulty speaking, I do not avoid communicating with people I know |  |  |  | **1** |
| dipd2 | My social life has not changed as a result of my dysarthria |  |  |  |  |
| dipd3 | I try other ways of getting my message across when people don’t understand me |  |  |  |  |
| dipd4 | I avoid using the telephone because of my speech |  |  |  | **1** |
| dipd5 | I avoid asking for items in shops |  |  |  |  |
| dipd6 | I rely on others to talk for me whenever possible |  |  |  |  |
| dipd7 | Because of my speech, I listen rather than take part in conversations |  |  |  |  |
| dipd8 | I do not avoid talking to strangers |  |  |  | **1** |
| dipd9 | I feel comfortable speaking in most situations both at home and outside |  |  |  |  |
| dipd10 | The difficulties I have with my speech restrict my social life |  |  |  | **1** |
| dipd11 | I only avoid talking when I am tired |  | **1** | **X** | **X** |
| dipd12 | Because of my speech I have become socially isolated |  | **1** | **X** | **X** |
| phif1 | My speech rate has changed | **1** | **X** | **X** | **X** |
| phif2 | My voice makes it difficult to express my emotions |  |  |  | **1** |
| phif3 | I have difficulty articulating when I speak |  | **1** | **X** | **X** |
| phif4 | I use a great deal of effort to speak |  |  |  |  |
| phif5 | I run out of air when I speak | **1** | **X** | **X** | **X** |
| phic1 | I have difficulties to express orally what I need (drink, eat, go to the restroom ...) |  |  |  |  |
| phic2 | I am hindered from expressing my thoughts, my opinions |  |  |  | **1** |
| phic3 | I have trouble communicating with unfamiliar people |  |  |  |  |
| phic4 | I am asked to repeat myself because of my difficulty to speak |  |  |  |  |
| phic5 | I speak with friends and neighbors or relatives less often because of my speech |  |  |  | **1** |
| phie1 | I suffer from my speech |  | **1** | **X** | **X** |
| phie2 | My speech difficulties limit my personal and social life |  |  |  | **1** |
| phie3 | I find other people don’t understand my speaking problem |  | **1** | **X** | **X** |
| phie4 | People seem irritated with my speech |  | **1** | **X** | **X** |
| phie5 | My speech makes me feel handicapped |  | **1** | **X** | **X** |
| phisev | What degree of severity do you give to your speech difficulties? | **1** | **X** | **X** | **X** |
| phigene | How difficult is it for you to produce understandable speech? |  | **1** | **X** | **X** |
| phihcp | How much does your speech limit your daily life? |  | **1** | **X** | **X** |
| eortc1 | Do you have any trouble doing strenuous activities, like carrying a heavy shopping bag or a suitcase? | **1** | **X** | **X** | **X** |
| eortc2 | Do you have any trouble taking a long walk? | **1** | **X** | **X** | **X** |
| eortc3 | Do you have any trouble taking a short walk outside of the house? | **1** | **X** | **X** | **X** |
| eortc4 | Do you need to stay in bed or a chair during the day? | **1** | **X** | **X** | **X** |
| eortc5 | Do you need help with eating, dressing, washing yourself or using the toilet? | **1** | **X** | **X** | **X** |
| eortc6 | Were you limited in doing either your work or other daily activities? | **1** | **X** | **X** | **X** |
| eortc7 | Were you limited in pursuing your hobbies or other leisure time activities? | **1** | **X** | **X** | **X** |
| eortc8 | Were you short of breath? | **1** | **X** | **X** | **X** |
| eortc9 | Have you had pain? | **1** | **X** | **X** | **X** |
| eortc10 | Did you need to rest? | **1** | **X** | **X** | **X** |
| eortc11 | Have you had trouble sleeping? | **1** | **X** | **X** | **X** |
| eortc12 | Have you felt weak? | **1** | **X** | **X** | **X** |
| eortc13 | Have you lacked appetite? | **1** | **X** | **X** | **X** |
| eortc14 | Have you felt nauseated? | **1** | **X** | **X** | **X** |
| eortc15 | Have you vomited? | **1** | **X** | **X** | **X** |
| eortc16 | Have you been constipated? | **1** | **X** | **X** | **X** |
| eortc17 | Have you had diarrhea? | **1** | **X** | **X** | **X** |
| eortc18 | Were you tired? | **1** | **X** | **X** | **X** |
| eortc19 | Did pain interfere with your daily activities? | **1** | **X** | **X** | **X** |
| eortc20 | Have you had difficulty in concentrating on things, like reading a newspaper or watching television? | **1** | **X** | **X** | **X** |
| eortc21 | Did you feel tense? | **1** | **X** | **X** | **X** |
| eortc22 | Did you worry? | **1** | **X** | **X** | **X** |
| eortc23 | Did you feel irritable? | **1** | **X** | **X** | **X** |
| eortc24 | Did you feel depressed? | **1** | **X** | **X** | **X** |
| eortc25 | Have you had difficulty remembering things? | **1** | **X** | **X** | **X** |
| eortc26 | Has your physical condition or medical treatment interfered with your family life? | **1** | **X** | **X** | **X** |
| eortc27 | Has your physical condition or medical treatment interfered with your social activities? | **1** | **X** | **X** | **X** |
| eortc28 | Has your physical condition or medical treatment caused you financial difficulties? | **1** | **X** | **X** | **X** |
| eortc29 | How would you rate your overall health during the past week? | **1** | **X** | **X** | **X** |
| eortc30 | How would you rate your overall quality of life during the past week? | **1** | **X** | **X** | **X** |
| hn31 | Have you had pain in your mouth? | **1** | **X** | **X** | **X** |
| hn32 | Have you had pain in your jaw? | **1** | **X** | **X** | **X** |
| hn33 | Have you had soreness in your mouth? | **1** | **X** | **X** | **X** |
| hn34 | Have you had a painful throat? | **1** | **X** | **X** | **X** |
| hn35 | Have you had problems swallowing liquids? | **1** | **X** | **X** | **X** |
| hn36 | Have you had problems swallowing pureed food? | **1** | **X** | **X** | **X** |
| hn37 | Have you had problems swallowing solid food? | **1** | **X** | **X** | **X** |
| hn38 | Have you choked when swallowing? | **1** | **X** | **X** | **X** |
| hn39 | Have you had problems with your teeth? | **1** | **X** | **X** | **X** |
| hn40 | Have you had problems opening your mouth wide? | **1** | **X** | **X** | **X** |
| hn41 | Have you had a dry mouth | **1** | **X** | **X** | **X** |
| hn42 | Have you had sticky saliva? | **1** | **X** | **X** | **X** |
| hn43 | Have you had problems with your sense of smell? | **1** | **X** | **X** | **X** |
| hn44 | Have you had problems with your sense of taste? | **1** | **X** | **X** | **X** |
| hn45 | Have you coughed? | **1** | **X** | **X** | **X** |
| hn46 | Have you been hoarse? | **1** | **X** | **X** | **X** |
| hn47 | Have you felt ill? | **1** | **X** | **X** | **X** |
| hn48 | Has your appearance bothered you? | **1** | **X** | **X** | **X** |
| hn49 | Have you had trouble eating? | **1** | **X** | **X** | **X** |
| hn50 | Have you had trouble eating in front of your family? | **1** | **X** | **X** | **X** |
| hn51 | Have you had trouble eating in front of other people? | **1** | **X** | **X** | **X** |
| hn52 | Have you had trouble enjoying your meals? | **1** | **X** | **X** | **X** |
| hn53 | Have you had trouble talking to other people? |  |  |  |  |
| hn54 | Have you had trouble talking on the telephone? |  |  |  | **1** |
| hn55 | Have you had trouble having social contact with your family? |  | **1** | **X** | **X** |
| hn56 | Have you had trouble having social contact with friends? |  | **1** | **X** | **X** |
| hn57 | Have you had trouble going out in public? |  | **1** | **X** | **X** |
| hn58 | Have you had trouble having physical contact with family or friends? | **1** | **X** | **X** | **X** |
| hn59 | Have you felt less interest in sex? | **1** | **X** | **X** | **X** |
| hn60 | Have you felt less sexual enjoyment? | **1** | **X** | **X** | **X** |
| hn61 | Have you used painkillers? | **1** | **X** | **X** | **X** |
| hn62 | Have you taken any nutritional supplements (excluding vitamins)? | **1** | **X** | **X** | **X** |
| hn63 | Have you used a feeding tube? | **1** | **X** | **X** | **X** |
| hn64 | Have you lost weight? | **1** | **X** | **X** | **X** |
| hn65 | Have you gained weight? | **1** | **X** | **X** | **X** |
| chiph1 | Do you have difficulty speaking? |  |  |  | **1** |
| chiph2 | Do people have difficulty understanding you? |  |  | **1** | **X** |
| chiph3 | Do you speak less with your family, friends, neighbours? |  |  | **1** | **X** |
| chiph4 | Do you have difficulty articulating? |  | **1** | **X** | **X** |
| chips1 | Is your personal and social life limited by problems related to your illness? | **1** | **X** | **X** | **X** |
| chips2 | Has your illness affected your relationship with other people? | **1** | **X** | **X** | **X** |
| chips3 | Are you bothered by problems related to your illness? | **1** | **X** | **X** | **X** |
| chips4 | Do you feel handicapped by your illness? | **1** | **X** | **X** | **X** |
|  |  |  |  |  |  |
|  | **TOTAL (24 items retained)** | **83** | **47** | **4** | **16** |
